# Photo-Crosslinked Silk Fibroin for 3D Printing

**DOI:** 10.3390/polym12122936

**Published:** 2020-12-09

**Authors:** Xuan Mu, Jugal Kishore Sahoo, Peggy Cebe, David L. Kaplan

**Affiliations:** 1 Department of Biomedical Engineering, Tufts University, Medford, MA 02155, USA; xuan.mu@tufts.edu (X.M.); Jugal.sahoo@tufts.edu (J.K.S.); 2 Department of Physics and Astronomy, Tufts University, Medford, MA 02155, USA; peggy.cebe@tufts.edu

**Keywords:** proteins, silk, additive manufacturing, photo-initiators, tyrosine, free radicals

## Abstract

Silk fibroin in material formats provides robust mechanical properties, and thus is a promising protein for 3D printing inks for a range of applications, including tissue engineering, bioelectronics, and bio-optics. Among the various crosslinking mechanisms, photo-crosslinking is particularly useful for 3D printing with silk fibroin inks due to the rapid kinetics, tunable crosslinking dynamics, light-assisted shape control, and the option to use visible light as a biocompatible processing condition. Multiple photo-crosslinking approaches have been applied to native or chemically modified silk fibroin, including photo-oxidation and free radical methacrylate polymerization. The molecular characteristics of silk fibroin, i.e., conformational polymorphism, provide a unique method for crosslinking and microfabrication via light. The molecular design features of silk fibroin inks and the exploitation of photo-crosslinking mechanisms suggest the exciting potential for meeting many biomedical needs in the future.

## 1. Introduction

**Figure 1:**
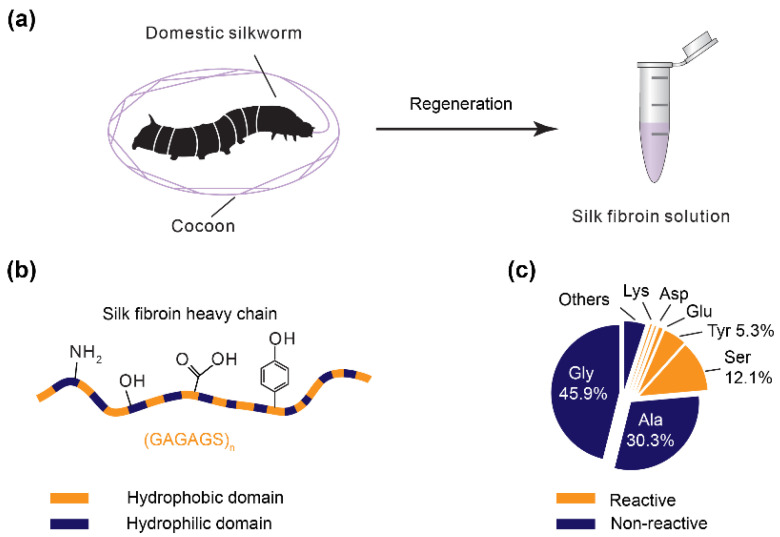
(**a**) Silk fibroin solution is regenerated from the cocoons of domestic *B. mori* silkworms. The regeneration often includes degumming and dissolution; (**b**) the silk fibroin heavy chain consists of alternating hydrophobic and hydrophilic domains. The hydrophobic domain is rich in the hexapeptide motif, GAGAGS, where G, A, and S are glycine, alanine, and serine, respectively. Some amino acid residues provide reactive groups for chemical modification and crosslinking, such as amine, hydroxyl, carboxyl, and phenol; (**c**) amino acid composition of the silk fibroin heavy chain. The percentage ratio is labeled unless it is less than one percent.

Silk fibroin is a protein regenerated from cocoons of the domestic silkworm *Bombyx mori* (*B. mori*) (Figure 1a) and has been broadly exploited for tissue engineering and regenerative medicine [1,2,3,4], bioelectronics [5,6,7] and bio-optics [8]. Silk fibroin (heavy chain) is a large protein with over 5000 amino acids and a molecular weight of around 390 kDa. Regenerated silk fibroin has a reduced molecular weight of around 100 kDa depending upon the specific degumming and dissolution conditions [9,10,11]. The majority of the amino acids in silk fibroin are glycine (45.9%) and alanine (30.3%), which show minimal chemical reactivity (Figure 1b,c) [12]. However, the less frequently occurring amino acids enable chemical reactions and crosslinking, including serine (12.1%), tyrosine (5.3%), threonine (0.9%), aspartic acid (0.5%), and glutamic acid (0.6%) [12]. 

The broad utility of silk fibroin is due to unique features compared with synthetic polymers and other protein-based materials, including biocompatibility, biodegradability, and host-implantation integration [13,14,15]. Furthermore, silk fibroin is available in large quantities from the textile industry and is relatively inexpensive in comparison to the recombinant production of proteins and harvesting proteins like collagen from mammalian tissues. The regeneration of silk fibroin into aqueous solutions enables processing versatility with the subsequent formation of distinct material formats, including fibers, films, sponges, and hydrogels [3,16]. Silk fibroin is also known to undergo directed assembly into mechanically superior materials, stronger than steel per density, and tougher than Kevlar [17]. The outstanding mechanical performance of these silk-based biomaterials results from both the hierarchical molecular architecture and β-sheet secondary structures [18,19,20]. The mechanical performance of silk-based materials is required to maintain the structural integrity of engineered tissue scaffolds and to mimic the biomechanical properties of native tissues, features that can be further tuned by process control using silk. In addition, silk fibroin has shown a degree of thermo-plasticity [18,21]. The β-sheet nanocrystals of silk fibroin can be melted under heating without degradation, which is promising for thermal processing of solid silk fibroin materials, analogous to widely used thermoplastics. All of the above features make silk fibroin an intriguing ink component for three-dimensional (3D) printing/additive manufacturing [2,22,23,24,25,26,27,28]. 

A key step in printing with silk fibroin solution is crosslinking, which connects dispersed, water-soluble silk fibroin chains into a continuous, water-insoluble network. Crosslinking methods for silk fibroin rely on either physical or covalent bonds. Silk fibroin can assemble into β-sheets, as physical crosslinks, which consist of tightly stacked segments between or within polypeptide chains via hydrogen bonds. The stacked segments are the crosslinks in the molecular network. The formation of β-sheets can be induced by salts [29,30,31], surfactants [32], heating [33], lyophilization [34], and organic solvents [35]. These β-sheets (crystals) usually endow rigid and strong mechanical properties to the silk materials, as the polypeptide chains are tightly stacked to form crystalline regions. Both aqueous salt baths [29] and methanol baths/treatment [36,37,38] have been developed for extrusion-based 3D printing with silk fibroin inks. Of note, both high salt concentration (>4 M) and organic solvents are harmful to mammalian cells, and thus are not amenable to printing with cell-laden inks.

In addition to β-sheet physical crosslinking, silk fibroin has been crosslinked covalently using enzymes, including horseradish peroxidase (HRP)/H_2_O_2_ [39,40] and tyrosinase [41]. These two enzymes oxidize the tyrosine residues to form di- or multi-tyrosine bonding as crosslinks. Tyrosinase-based crosslinking often requires hours; by contrast, HRP/H_2_O_2_ crosslinking is relatively fast, in the range of tens of minutes [39,40]. The enzyme-crosslinking often renders silk fibroin hydrogels with considerable elasticity, compared with the stiffer gels formed by physical crosslinking via β-sheets, and has been used for 3D printing [42,43]. However, enzyme-crosslinking is limited, as the dynamics of enzyme-crosslinking can be challenging to control. This limitation can result in poor control of the shape of silk fibroin hydrogels and the applicable range of silk fibroin concentration [44]. Furthermore, exogenous enzymes after crosslinking can be entrapped in the hydrogels, as the mesh size of the molecular networks is small, and this could have an adverse impact on cells and elicits a harmful immune response unless human-derived peroxidase is used [45]. The Fenton reaction is another option to covalently crosslink silk fibroin via dityrosine bonds and is promising for 3D printing [46,47].

**Figure 2:**
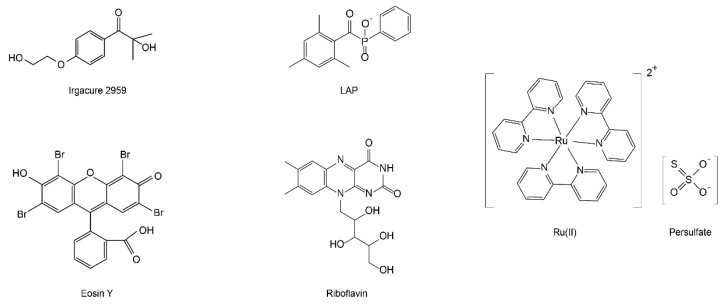
Common photoinitiators for crosslinking either native or chemically modified silk fibroin.

Photo-based crosslinking, in contrast to β-sheet and enzyme-based approaches, provide unique advantages for 3D printing with silk fibroin inks. The crosslinking dynamics are conveniently controlled by light intensity via tuning distance and time. Laser scanning and projected light allow for fine printing resolution (i.e., printed filaments down to sub-micrometer diameter), leading to well-controlled complex shapes for the silk fibroin hydrogels. Several photo-crosslinking mechanisms are applicable to native silk fibroin and chemically modified versions, including photo-oxidation of the tyrosine residues and free radical polymerization of methacryloyl groups. A range of photo-initiators can be selected according to distinct spectral features and crosslinking efficiency, including 2-hydroxy-1-[4-(2-hydroxyethoxy) phenyl]-2-methyl-1-propanone (Irgacure 2959) [48], lithium acylphosphinate salt (LAP) [48,49], tris(2,20-bipyridyl) dichlororuthenium(II) (Ru(II))/persulfate [43,50,51], eosin Y [52], rose bengal [53], and riboflavin [54] (Figure 2 and Table 1). The light used to excite these photoinitiators is important for applications that involve cells. Ultraviolet (UV) light usually shows high crosslinking efficiency due to higher light energy compared with visible light; however, UV light is harmful to cells, especially the nucleus [55]. In contrast, visible light enables cytocompatible processing conditions. 

In this review, we introduce general photo-crosslinking mechanisms that have been exploited for 3D printing silk fibroin-based inks, mainly including photo-oxidation, and methacryloyl-based free radical polymerization. We also demonstrate the conformational polymorphism of silk proteins that has been exploited for fabricating microstructures via light. Advanced light-assisted 3D printing techniques with silk fibroin inks would have a broad range of implications in biomedicine and beyond. 

## 2. Photo-Oxidization

Photo-oxidization involves the formation of oxygen radicals upon exposure to light, followed by a reduction-oxidation reaction. Some amino acids demonstrate considerable redox properties, such as tyrosine and tryptophan [56], with the oxidative coupling of two adjacent residues [50,57]. Because of the high content and distribution of tyrosine (5.4%), silk fibroin is particularly suitable for photo-oxidization. Tyrosine in silk fibroin is reportedly present at the boundary of crystalline-forming regions. Photo-oxidization efficiency is also associated with the overall content and special distribution of the redox amino acids [57].

### 2.1. Ru(II)/Persulfate

**Figure 3:**
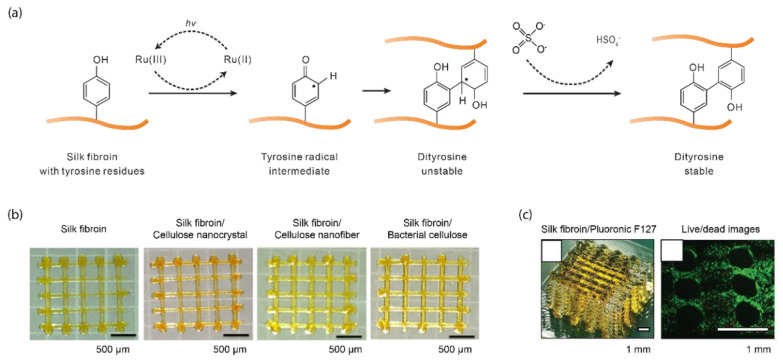
Ru(II)/persulfate-mediated photo-crosslinking of silk fibroin. (**a**) Ru(II) and persulfate, under light exposure, are photolyzed into Ru(III) and sulfate radicals, respectively. Ru(III) oxidizes tyrosine residues into a tyrosyl radical intermediate. The tyrosyl intermediate couples with nearby tyrosine residues, which are stabilized by the persulfate radical by removing a hydrogen atom. (**b**) Silk fibroin is blended with nanocellulose materials for 3D printing via extrusion and Ru(II)/persulfate. Reproduced with permission from Reference [70] Copyright 2020 American Chemistry Society. (**c**) Three-dimensional lattice silk fibroin prints made by sacrificial Pluronic F127 template and Ru(II)/persulfate. The live and dead cell assay shows cellular viability after one-day in culture. Reproduced with permission from Reference [51] Copyright 2020 John Wiley and Sons.

Ru(II)/persulfate has been widely used for photo-polymerization via tyrosine residues in silk fibroin [51,58,59,60], and other proteins, including resilin [61,62,63,64], gelatin [65,66], and fibrin [67]. Ru(II) has a high molar attenuation coefficient of 14,600 M^−1^ cm^−1^ at 450 nm, which supports the use of visible light, especially blue light, where Ru(II) and persulfate are photolyzed into Ru(III) and sulfate radicals, respectively (Figure 3a). Both sodium and ammonium persulfate are commonly used without a noticeable difference in the efficiency of photo-crosslinking. Ru(III) oxidizes tyrosine residues into a tyrosine radical intermediate that forms arene to couple with nearby tyrosine groups via the formation of dityrosine. The dityrosine is further stabilized by the removal of a hydrogen atom via a persulfate radical. The biocompatibility of ruthenium has not been extensively studied [41]. Ru(II) is not a structural component of the crosslinked polymer network and can be extracted, mitigating potential biohazards. Nevertheless, in the in vitro culture of human chondrocytes, Ru(II) was cytocompatible up to 20 mM, exceeding the concentration used for photo-crosslinking (2 mM) [68,69]. Similarly, sodium persulfate shows no cytotoxicity up to 50 mM [69]. Sodium persulfate at 20 mM for less than one hour is suggested for cytocompatibility [68]. 

Ru(II)/persulfate was employed to crosslink silk fibroin in a facile and rapid manner [58]. The resultant silk fibroin hydrogels showed a low water uptake (0.7 g/g, the ratio of the mass of water to the mass of dry weight) compared with that of recombinant resilin gels (2–4 g/g), partly due to the hydrophobic sequences of silk fibroin. The water uptake was also used to estimate the crosslink density that approached 0.4 g/g using a modified Florye–Rehner equation [71]. According to Fourier transform infrared (FTIR) spectra of amide I band, the photo-crosslinked hydrogels showed a higher content of β-sheet (48%) than other secondary structures, such as β-turns (37%) and random coils (19%), indicating the dominance of β-sheets in the hydrogel. Using differential scanning calorimetry (DSC), and dynamic mechanical analysis (DMA), the hydrogels also showed an increased storage modulus (E’) and tan (δ) along with the decreased level of hydration. δ is the phase angle shift between loss modulus (E’’) and the storage modulus (E’). Tan (δ) is a damping factor and indicates the energy absorbed or dissipated in the process of deformation, the opposite of resilience. This result indicates that the elastic properties of the photo-crosslinked silk fibroin hydrogel are related to the level of hydration. 

A range of functional additives can be used to enhance the mechanical performance of the Ru(II)/persulfate-mediated photo-crosslinked silk fibroin hydrogels, including resilin, graphene oxide (GO), and cellulose [59,70,72,73]. Resilin is one of the most resilient (~92%) proteins, responsible for the jumping and flight of many insects [62,74]. Resilin also has a high content of tyrosine (~6.9%), enabling the efficient formation of dityrosine crosslinks. Composite hydrogels of silk fibroin and resilin demonstrated an improved water uptake (0.9 g/g), compared with monolithic silk fibroin hydrogels (0.4 g/g) [58], which leads to improved elasticity. However, β-sheets (43%) still dominate, compared with other secondary structures, such as β-turns (36%) and random coils (21%). Another additive, GO, is widely used to modify the mechanical properties of silk fibroin-based materials [75]. A photo-crosslinked composite hydrogel of silk fibroin and GO demonstrated improved tensile mechanical properties, such as Young’s modulus and extensibility [72]. The Young’s modulus was around 8 MPa, higher than monolithic silk fibroin hydrogels (1 MPa) and native cartilage (1.5 MPa); the ultimate tensile strain was around 8%, higher than monolithic silk fibroin hydrogel (3.25%). The toughness of the silk fibroin/GO composite hydrogel was around 2.4 MJ m^−3^, 2.5 times higher than that of monolithic silk hydrogels. Despite the widespread use of GO to enhance mechanical performance, its toxicity may compromise the biocompatibility of silk fibroin. The toxicity of GO is often associated with the size and surface coatings [76]. Other investigations have found that GO with a dose less than 20 µg/mL and 1 mg/kg body weight shows no obvious toxicity to human fibroblasts [77] or to mice [78].

Cellulose is sustainable and offers superior mechanical strength [70] and has been used as a useful additive for 3D printing [84]. The addition of bacterial cellulose dramatically increased the toughness of photo-crosslinked silk-cellulose composites to 108 kJ m^−3^, six-fold higher than monolithic silk fibroin hydrogels (around 16.7 kJ m^−3^). These silk fibroin/nanocellulose composites can be 3D printed into a two-layer rectangle lattice (Figure 3b). These results highlight the general strategy of using structural additives to improve the mechanical properties of photo-crosslinked silk fibroin hydrogels. 

A poloxamer Pluronic F127 (30 wt%) was used to improve the 3D printability of silk fibroin crosslinked by the Ru(II)/persulfate (Figure 3c) [51]. Pluronic F127 gels at room temperature (25 °C) and liquefies at a lower temperature (4 °C), useful as a sacrificial template in 3D printing [85]. At high concentrations such as 30 wt%, Pluronic F127 is more viscous than silk fibroin solution (5 wt%) and can maintain the shape of extruded filaments. A 3D structure in Pluronic F127 was first printed, followed by printing silk fibroin solution (containing Ru(II) and persulfate). Through capillary forces, the printed silk fibroin infiltrates into the void space within the Pluronic F127 prints; this composite print of silk fibroin and Pluronic F127 was then photo-crosslinked, followed by incubation at 4 °C for 3 min to remove Pluronic F127. Largely due to the Pluronic F127, the printing resolution of silk fibroin was improved down to 40 µm. Human articular chondrocytes, encapsulated within these silk fibroin prints, retained cell viability, 72% (±1), and secreted cartilage-specific matric components, including glycosaminoglycans, collagen type I, collagen type II, and aggrecan [51].

### 2.2. Riboflavin

In addition to the Ru(II)/persulfate, riboflavin provides another important photo-oxidation approach for crosslinking silk fibroin [54,79]. Riboflavin is a nutrient, vitamin B2, found in native tissues; its absorbance peak ranges from 350 to 450 nm, allowing excitation via visible light. However, the solubility of riboflavin in aqueous solutions is low (0.045 mg/mL) [86], limiting the photo-crosslinking kinetics [48]. Thus, a derivative of riboflavin, flavin mononucleotide with high solubility in aqueous solutions (92 mg/mL) is frequently used. The extinction coefficient of riboflavin (15,800 M^−1^ cm^−1^) [87] is slightly higher than that of Ru(II) (14,600 M^−1^ cm^−1^) [88], both at 450 nm, implying comparable capability of the two photoinitiators to absorb light. Riboflavin-based photo-crosslinking is particularly promising for cell-involved applications.

**Figure 4:**
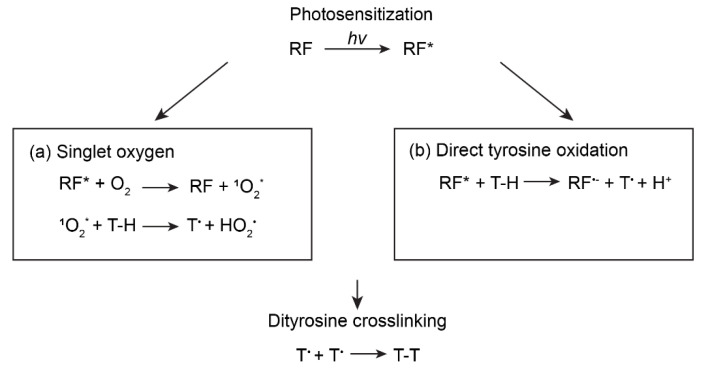
Photo-crosslinking mechanisms of riboflavin (RF). Upon light exposure, RF becomes photo-sensitized (RF*). (**a**) RF* reacts with dissolved oxygen to generate singlet oxygen (^1^O_2_*) that further gives rise to tyrosine radicals (T^•^). Reproduced with permission from Reference [92]. Copyright 2020 Elsevier. (**b**) RF*can directly react with tyrosine residue (T–H) to produce T^•^. Reproduced with permission from Reference [54]. Copyright 2016 John Wiley and Son. In both mechanisms, T^•^ reacts with another one via radical–radical termination to form covalent dityrosine crosslinks.

Riboflavin-mediated photo-crosslinking is largely subject to the same mechanism as Ru(II)/persulfate, where the crosslinking of silk fibroin is achieved by the oxidation of tyrosine residues and the formation of di-tyrosine bonds. However, two mechanisms of riboflavin-mediated oxidation of tyrosine have been proposed: singlet oxygen [79,89] and direct oxidization [54,90] (Figure 4). In the first mechanism, photo-sensitized riboflavin reacts with dissolved oxygen to generate singlet oxygen (^1^O_2_), which tends to produce reactive oxygen species (ROS), such as peroxides (O–O) [79]. The ROS oxidizes reductive amino acid residues, such as tyrosine, to form tyrosyl radicals. The other mechanism proposes that photo-sensitized riboflavin directly reacts with tyrosine residues, which gives rise to tyrosyl radicals. In both mechanisms, the tyrosyl radicals react with each other to form the dityrosine crosslinks [91]. To investigate the possible mechanism, sodium azide, a radical oxygen scavenger, was added to the precursor solution of silk fibroin and riboflavin [54]. If radical oxygen is involved in the crosslinking, the sodium azide would scavenge the radical oxygen and disrupt crosslinking; indeed, the sodium azide decreased the storage modulus of the hydrogel. However, the storage modulus was still higher than negative controls in the absence of riboflavin or light, indicating that hydrogel crosslinking still exists, perhaps due to either undepleted radical oxygen or photo-sensitized riboflavin-induced tyrosyl radicals. Thus, neither of the two mechanisms can be excluded. The photo-crosslinked silk fibroin hydrogel via riboflavin showed elasticity compared with enzymatically crosslinked examples [79]. The hydrogels showed a compressive strength of around 50 kPa and a compressive resilience of 90% (area ratio of loading and unloading curves), depending on the concentration of silk fibroin used in the reaction. 

The use of riboflavin alone for crosslinking silk fibroin is slow, taking about one hour for the formation of a thin film [54]. The slow crosslinking dynamics are unfavorable for 3D printing. The addition of HRP [79] and sodium persulfate [68] has been suggested to accelerate the gelation by continuously boosting the generation of free radicals. By increasing the concentration of sodium persulfate from 10 to 40 mM, the gelation time decreased from 6.53 ± 0.43 to 4.28 ± 0.33 min [68]. An effect similar to that of sodium persulfate on accelerating photo-crosslinking has also been found with other proteins, such as keratin [93]. The gelation time of keratin hydrogel formation decreased from 20 to 5 min upon the increase in sodium persulfate concentration from 70 to 160 mM [93]. 

## 3. Methacryloyl-Modification

Besides intrinsic tyrosine residues, silk fibroin has been chemically modified with other functional groups, such as methacryloyl, for photo-crosslinking. The methacryloyl group allows free-radical polymerizations that are highly efficient and rapid. Methacryloyl modification has been a general strategy to photo-crosslink resins [94] and naturally-derived polymers, such as gelatin [95,96,97,98], hyaluronic acid [99], and chitosan [100].

**Figure 5:**
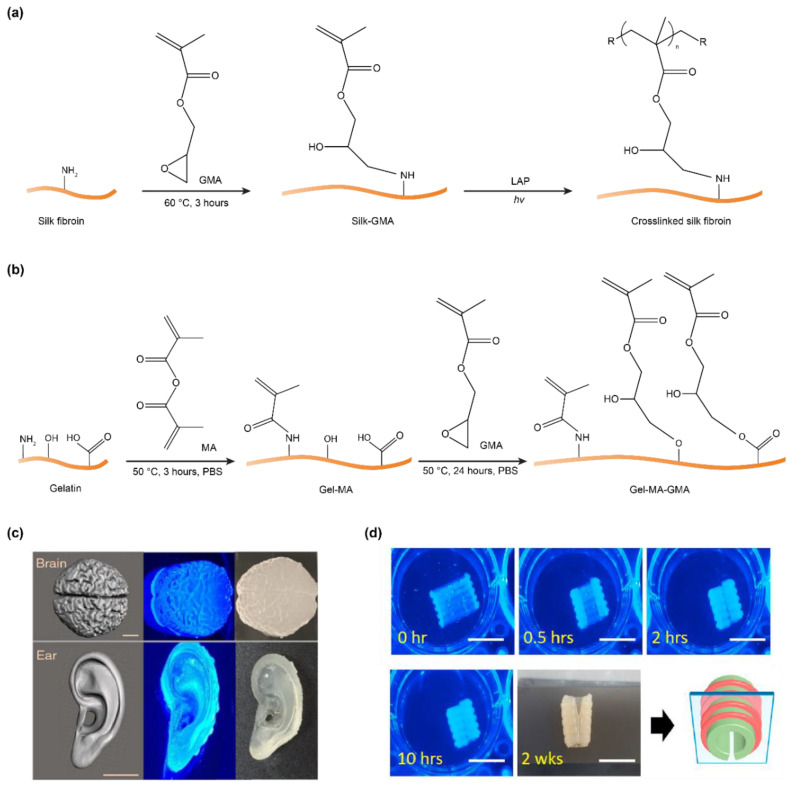
(**a**) Schematics of methacryloyl-modification of silk fibroin via glycidyl methacrylate (GMA) and photo-crosslinking via lithium acylphosphinate salt (LAP). The illustration shows only one methacryloyl substitution on the amine group for clarity. It is possible to modify the amine group with two methacryloyl substitutions [49]. (**b**) Gelatin was modified with methacryloyl groups at amine, hydroxyl, and carboxyl groups in a two-step manner [104]. (**c**) Three-dimensionally printed brain- and ear-like silk fibroin structures via digital light processing (DLP). Scale bars, 1 cm. Reproduced with permission from Reference [49] under the Creative Commons Attribution License. (**d**) Three-dimensionally printed planar silk fibroin structures that transform into C-shape as a scaffold for repairing the trachea. Scale bars, 1 cm. Reproduced with permission from Reference [80]. Copyright 2020 Elsevier.

Methacryloyl substitution can occur at the amine, hydroxyl, and carboxyl groups, which are available in silk fibroin (Figure 1b,c), depending on specific reaction conditions and modification reagents [101,102]. Both methacrylic anhydride (MA) and glycidyl methacrylate (GMA) have been used to introduce methacryloyl groups. However, the two chemicals were found to have different effects on silk fibroin [49]. MA leads to premature crystallization of silk fibroin and thus compromises the efficiency of the reaction. This result is ascribed to a by-product, methacrylic acid, that reduces the pH and induces the crystallization of silk fibroin. In contrast, GMA modification primarily undergoes an epoxide ring-opening that results in no acidic products, thus facilitating methacryloyl modification. The degree of methacryloyl substitution is associated with the concentration of GMA; 424 mM showed the highest degree of crosslinking density at 42%. GMA is proposed to react with the primary amines of lysine residues, around 0.2% of all amino acids of silk fibroin, and was confirmed by proton nuclear magnetic resonance (^1^H NMR) [49,103] (Figure 5a). However, GMA is reported to react with other groups, such as hydroxyl and carboxyl, via both the transesterification and the epoxide ring-opening mechanisms [102,104] (Figure 5b). For silk fibroin heavy chains, the amino acid residues with hydroxyl and carboxyl groups have a higher molar ratio (around 19.4%) than those with amine groups (lysine, 0.2%) [105,106,107]. Thus, methacryloyl substitution at the hydroxyl and carboxyl groups may increase the number of cross-linkable sites and thus the photo-crosslinking density of silk fibroin. Indeed, this strategy has been used to improve the photo-crosslinking density of gelatin methacryloyl (GelMA) for prolonging degradation and enhancing mechanical strength [104].

Methacryloyl-substituted silk fibroin (silk-GMA) has been used in digital light processing (DLP)-based 3D printing [49,80,81]. DLP requires around one second to print 1 mm^3^ with a resolution of 1 µm; the fast printing of DLP is due to the projection of a whole cross-sectional layer, in contrast to the point scanning in other light-assisted 3D printing, such as stereolithography. The 3D printed Silk-GMA structures, due to enhanced mechanical strength, allows tensile testing that is often challenging for many silk fibroin hydrogels. The ultimate tensile strength and strain of 30% silk-GMA was 75 kPa (±7.5) and 124.2% (±41), respectively, which is higher than that (52 kPa (±4.3) and 77.6% (±3.8)) of 20% silk-GMA [49]. This result indicates a strategy to enhance tensile mechanical properties by increasing the concentration of silk-GMA, which leads to more crosslinks and entanglements among the silk macromolecules. The silk-GMA can be printed into complex shapes to mimic tissues, such as the brain and ear (Figure 5b) [49]. The printed tissue-like structures are elastic and are able to undergo reversible compression.

Three-dimensionally printed silk-GMA (30 w/v%) also showed a compressive strength of 910 ± 127 kPa and compressive modulus of 125.8 ± 34 kPa, which is higher than either polycaprolactone (PCL)-blended gelatin hydrogels (75–94 kPa) or 30% GelMA (88 kPa) [49]. The compressive properties of silk-GMA are promising for cartilage tissue engineering [81]. In addition, silk fibroin has shown the capability to promote the proliferation and differentiation of chondrocytes [108]. The 3D-printed silk-GMA hydrogel enables the encapsulation of human cells for up to four weeks for chondrogenesis. Furthermore, in vivo experiments of the cell-laden silk-GMA show the capability to repair partial defective rabbit trachea by forming collagen-based matrices and epithelium, which were characterized by Masson’s trichrome staining and hematoxylin/eosin staining, respectively [81]. 

The 3D-printed silk-GMA was exploited for constructing a shape-morphed tissue scaffold originating from anisotropic swelling ratios between top and bottom layers [80]. The distinct swelling ratio was ascribed to the difference in surface area. As a result, a planar sheet-like silk-GMA was transformed into a C-shaped trachea scaffold (Figure 5c). Within the scaffold, human turbinate-derived stem cells and human chondrocytes were encapsulated in different layers to mimic the mucus membrane and hyaline cartilage ring, respectively [80]. Of note, the C-like shape was particularly advantageous for pediatric patients, as it fits the growing airway by expanding [109]. The shape morphing capability of protein-based materials has also been observed with bovine serum albumin [110,111], calmodulin [112], and silk-elastin-like proteins [113], pointing to the development of 3D printing with shape-morphing protein-based materials.

## 4. Conformational Polymorphism

Silk fibroin has polymorphic conformations that have distinct water-solubility, including the water-soluble random coils and water-insoluble β-sheets. The polymorphic conformation is due to the molecular design of silk fibroin that contains alternating hydrophobic and hydrophilic domains (Figure 1b), and the β-sheet mainly consists of the hexapeptide motif, GAGAGS, rich in the hydrophobic domains. The polymorphic conformations also play roles in natural silk spinning, where aqueous silk solution is stored in the spinning gland largely with random coil conformation; the spun silk fiber is a water-insoluble solid with predominately β-sheet conformation [2]. 

**Figure 6:**
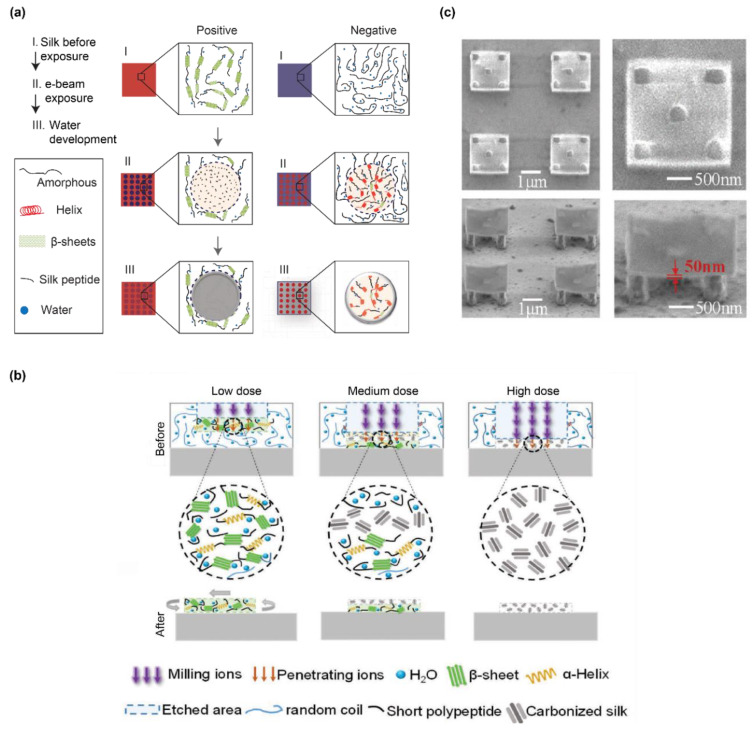
(**a**) Polymorphic conformation of silk fibroin has been employed for positive and negative lithography via electron beam. Reproduced with permission from Reference [115]. Copyright 2014 Springer Nature. (**b**) Dose of ion beam leads to different conformational transitions and structures. Reproduced with permission from Reference [117]. Copyright 2018 John Wiley and Sons. (**c**) SEM images of 3D desks with four pillars via the combination of ion and electron beams. Reproduced with permission from Reference [117]. Copyright 2018 John Wiley and Sons.

The conformation-dependent water solubility of silk fibroin has been exploited for electron-beam lithography that leads to 2D silk structures [114,115]. Depending on the initial conformation, silk fibroin undergoes either positive or negative lithography (Figure 6a). For silk fibroin with predominate β-sheet conformation, the exposure of the electron beam leads to thermal degradation or cleavage of the polypeptide chains, leading to void space after water rinsing. In contrast, for silk fibroin with predominately random coil conformation, the electron beam results in the formation of β-sheets and water-insoluble silk structures. Other unexposed silk fibroin remains in a random coil conformation and dissolves in water and is removed after the water extraction. One way to improve the pattern resolution and surface roughness of the patterned silk fibroin structures is to use silk fibroin light chains; in contrast to the heavy chain, the regenerated light chain has both low molecular weight and a narrow molecular weight distribution [116]. The resolution and roughness have been improved to 1.6 µm and 2 nm, respectively, in comparison to 2.5 µm and 40 nm with the use of the silk fibroin heavy chain (after 10 min of degumming) [116]. 

Conformational polymorphism is also exploited for recombinant spider silk proteins patterned by ion beam lithography [117] (Figure 6b). Given the similar conformational polymorphism between spider silks and silk fibroin, light-assisted fabrication for one protein is applicable to the other. The dose of the ion beam is associated with the conformation and structures of the silk proteins. At a low dose of the ion beam, only silk proteins near the solution surface are crosslinked by β-sheet formation; at a medium dose, the silk proteins near the surface and near the bottom are carbonized and crosslinked, respectively, leading to water-insoluble silk structures anchored on the bottom surface; finally, at a high dose, all silk proteins exposed to the ion beam are carbonized, resulting in the formation of void space. Furthermore, the ion beam was combined with an electron beam to make a complex 3D desk structure at the nanoscale in two steps [117] (Figure 6c). In the first step, the electron beam was used to form nanopillars from the bottom surface; in the second step, the ion beam was used to form the surface of the desk using the low dose.

## 5. Double Crosslinking

The photo-crosslinking of silk fibroin can be combined with other crosslinking mechanisms, such as the physical crosslinking of the β-sheets [118]. This strategy of double crosslinking [96] is promising to enhance mechanical properties by increasing crosslink density and introducing crystalline regions. A Ru(II)/persulfate-crosslinked silk fibroin hydrogel was treated with methanol to form β-sheet crosslinks [60]. Due to the double-network, this hydrogel demonstrated compressive moduli of ~1.3 and ~11 MPa at 20% and 40% strain, respectively. This magnitude of modulus was higher than many native tissues (articular cartilage, 0.3–0.8 MPa and skin, 5.7 kPa) and synthetic hydrogels (0.1–1 MPa) [119]. The utility of this hydrogel was demonstrated in the encapsulation of the enzyme carbonic anhydrase, which remained 60% active. A 3D-printed silk fibroin hydrogel generated via riboflavin-mediated photo-crosslinking formed β-sheets in phosphate-buffered saline solution at 37 °C [120]. However, the emergent β-sheet led to shrinkage in size. The addition of gelatin was proposed to mitigate the shrinking and improve printing accuracy [120].

Double crosslinking of silk fibroin has also been found in multiphoton lithography (MPL) [121,122]. MPL allows direct writing of high-resolution 3D structures in a mask-free manner [123,124]. MPL-patterned silk fibroin usually features small filaments down to hundreds of nanometers and overhanging structures. Silk fibroin was blended with methylene blue for photo-crosslinking via a femtosecond laser into various 3D structures [122]. The silk structures possessed both β-sheets and dityrosine bonds. The β-sheets, characterized by FTIR, were ascribed to the temperature of 70–100 °C due to the laser irradiation. In another study, the β-sheet that dominated the conformation of MPL-patterned silk fibroin hydrogels was suggested by electron diffraction patterns in conjunction with transmission electron microscopy (TEM) analysis [121]. The dityrosine bond of the MPL-patterned silk fibroin hydrogel was attributed to the photo-oxidation of photoinitiators, such as methylene blue, Ru(II), or rose bengal, and confirmed by FTIR, fluorescence, and insolubility at a 60 °C, 9.3 M LiBr solution. The LiBr solution can dissolve β-sheets by disrupting hydrogen bonds, yet is incapable of disrupting the covalent dityrosine crosslinks.

## 6. Conclusions

Silk fibroin has been increasingly exploited as a potent component for formulating 3D printing inks. The proteinaceous composition makes silk fibroin biologically relevant and capable of building scaffolds with desired cytocompatibility, bio-degradability, and immune tolerance. These properties are often challenging for synthetic polymers. Photo-crosslinking provides considerable fabrication versatility with silk fibroin. The rapid crosslinking time and well-controlled projection area enable rapid and high-resolution 3D printing. A series of photo-crosslinking mechanisms have been applied to silk fibroin with distinct advantages and limitations. Photo-oxidation is advantageous in maintaining the protein structure and eliminating the need for chemical modification. In contrast, the methacrylate free radical polymerization requires chemical modification and, often, the use of organic solvents, such as acetone and ethanol, yet is better in terms of photo-crosslinking efficiency. More importantly, photo-crosslinked silk fibroin has been successfully translated into a variety of 3D printing/additive manufacturing techniques. A range of photo-crosslinked silk fibroin prints, such as scaffolds for trachea and bone, have shown anatomical accuracy and cytocompatibility, and thus are promising in meeting clinical needs.

There are several directions to promote the use of photo-crosslinked silk fibroin for 3D printing. Translating biological manufacturing principles, i.e., hierarchical molecular assembly, into photo-assisted processing and 3D printing is one goal. Such biomimetic 3D printing is promising for building strong artificial structures with protein molecules. Besides exploiting new photo-crosslinking mechanisms, computational simulation has been increasingly used to understand underlying biological manufacturing principles [125,126], implying a crucial role in developing 3D printing techniques with protein inks. Exploiting new additives to improve the printability and mechanical performance of photo-crosslinked silk fibroin prints is also a priority. We have shown that additives and double crosslinking can enhance mechanical performance. However, many of the present additives may compromise the advantages of monolithic silk fibroin inks. Despite substantial challenges ahead, photo-crosslinked silk fibroin holds considerable promise for 3D printing.

## Figures and Tables

**Table 1:** Summary of photo-crosslinking conditions of silk fibroin and other proteins.

Proteins	Crosslinking Sites	Solution/Ink (mg/mL)	Photoinitiators	Light Source	Exposure Distance	Exposure Time	Refs
**Silk fibroin**	Tyrosine (~5.3 mol%) ^a^	75 (+100 Resilin)	Ru(II), 5 mM APS, 28 mM	250 W white	N/A	120 + 60 s (Two sides)	[59]
		~300	Ru (II), 0.16–10 mM APS, 20, 28, 100 mM	250 W white	N/A	120 + 60 s (Two sides)	[58]
		~20	Ru(II), 0.5 mM APS, 5 mM	400–450 nm 30 mW/cm^2^	N/A	3 min	[51]
		10–50	Riboflavin (0.1 mM) HRP (10 U/mL)	365 nm 300 W/m^2^	N/A	30 min	[79]
		50	Riboflavin 5′-monophosphate, 2 mM	450 nm (×3 leds) 18.7 mW/cm^2^	N/A	~60 min	[54]
	Methacryloyl	100–300	LAP, 0.2%	365 nm 30 mW/cm^2^	N/A	N/A	[49]
		100–300	LAP, 0.2%	365 nm 3.5 mW/cm^2^	N/A	~5 s (each layer)	[80]
		100–300	LAP, 0.6%	365 nm	N/A	~5 s (each layer)	[81]
**Gelatin/Collage**	Tyrosine (~0.9%) ^a^	10, Gel-MA/ 0.6, Collagen	Ru(II), 0.2–2 mM APS, 2–20 mM	400–450 nm 3–100 mW/cm^2^	N/A	15 min	[82]
		100–175, gelatin	Ru(II), 1 mM SPS, 20 mM	600 W white	150 mm	30 s	[65]
**Fibrin**	Tyrosine (4.9%, β-chain; 5.6%, γ-chain; 0.65%, α-chain)	3	Ru(II), 2 mM SPS, 43 mM	458 nm 28 mW/cm^2^	30 mm	10 s	[67]
		100, 150	Ru(II), 2 mM SPS, 20 mM	600 W white	150 mm	20 s	[69]
**GB1-resilin polyprotein**	Tyrosine (~6%) ^a^	200	Ru(II), 0.2 mM APS, 50 mM	200 W white	N/A	30 s	[64]
**Rec1-resilin**	Tyrosine (~6%) ^a^	200	Ru(II), 0.2–2 mM APS, 10 mM	600 W white	150 mm	20 s	[62]
**Mussel adhesive proteins**	Tyrosine (~20%) ^a^	100–300	Ru(II), 2 mM SPS, 10–30 mM	460 nm 1200 mW/cm^2^	20 mm	60 s	[83]

Ru(II), tris(2,2-bipyridyl)dichlororuthenium(II) hexahydrate; LAP, lithium phenyl (2,4,6-trimethylbenzoyl) phosphinate; SPS, sodium persulfate; APS, ammonia persulfate; Gel-MA, methacryloyl gelatin; HA, hyaluronic acid; HRP, horseradish peroxidase; N/A, not applicable; ^a^ molar ratio of amino acid residues.
